# Concordance of the treatment patterns for major depressive disorders between the Canadian Network for Mood and Anxiety Treatments (CANMAT) algorithm and real-world practice in China

**DOI:** 10.3389/fphar.2022.954973

**Published:** 2022-08-31

**Authors:** Lu Yang, Yousong Su, Sijia Dong, Tao Wu, Yongjing Zhang, Hong Qiu, Wenjie Gu, Hong Qiu, Yifeng Xu, JianLi Wang, Jun Chen, Yiru Fang

**Affiliations:** ^1^ Clinical Research Center and Division of Mood Disorders, Shanghai Mental Health Center, Shanghai Jiao Tong University School of Medicine, Shanghai, China; ^2^ Global Epidemiology, Office of Chief Medical Officer, Johnson & Johnson, Shanghai, China; ^3^ Global Epidemiology, Office of Chief Medical Officer, Johnson & Johnson, Beijing, China; ^4^ Global Epidemiology, Office of Chief Medical Officer, Johnson & Johnson, Titusville, NJ, United States; ^5^ Departments of Community Health and Epidemiology, Faculty of Medicine, Dalhousie University, Halifax, NS, Canada; ^6^ CAS Center for Excellence in Brain Science and Intelligence Technology, Shanghai, China; ^7^ Shanghai Key Laboratory of Psychotic Disorders, Shanghai, China

**Keywords:** depressive disorder, treatment patterns, guideline adherence, treatment algorithm, real-world studies

## Abstract

**Background:** Antidepressant (AD) algorithm is an important tool to support treatment decision-making and improve management of major depressive disorder (MDD). However, little is known about its concordance with real-world practice. This study aimed to assess the concordance between the longitudinal treatment patterns and AD algorithm recommended by a clinical practice guideline in China.

**Methods:** Data were obtained from the electronic medical records of Shanghai Mental Health Center (SMHC), one of the largest mental health institutions in China. We examined the concordance between clinical practice and the Canadian Network for Mood and Anxiety Treatments (CANMAT) algorithm among a cohort composed of 19,955 MDD patients. The longitudinal characteristics of treatment regimen and duration were described to identify the specific inconsistencies. Demographics and health utilizations of the algorithm-concordant and -discordant subgroups with optimized treatment were measured separately.

**Results:** The overall proportion of algorithm-concordant treatment significantly increased from 84.45% to 86.03% during the year of 2015–2017. Among the patients who received recommended first-line drugs with subsequent optimized treatment (*n* = 2977), the concordance proportion was 27.24%. Mirtazapine and trazodone were the most used drugs for adjunctive strategy. Inadequate or extended duration before optimized treatment are common inconsistency. The median length of follow-up for algorithm-concordant (*n* = 811) and algorithm-discordant patients (*n* = 2166) were 153 days (Q1-Q3 = 79–328) and 368 days (Q1-Q3 = 181–577) respectively, and the average number of clinical visits per person-year was 13.07 and 13.08 respectively.

**Conclusion:** Gap existed between clinical practice and AD algorithm. Improved access to evidence-based treatment is required, especially for optimized strategies during outpatient follow-up.

## Introduction

Major depressive disorder (MDD) is one of the most common psychiatric disorders and a leading disease burden worldwide due to its disabling nature and recurrent course (World Health Organization, 2020). Despite advances in the understanding of neuropharmacology, the treatment outcomes of depression were unfavorable. Over half of the patients with MDD failed to respond satisfactorily to the initial pharmacotherapy (Emslie et al., 2009; Rossom et al., 2016). In the first phase of Sequenced Treatment Alternatives to Relieve Depression (STAR*D) trial, 53% of patients showed non-response to a 14-week monotherapy with citalopram (Trivedi et al., 2006). Those with inadequate response would suffer from a high risk of disease progression, recurrence, and impaired function (Bergfeld et al., 2018; Kraus et al., 2019). Increasing treatment steps along with optimized pharmacological strategies were often required to achieve sufficient response (Tundo et al., 2015; Bayes and Parker, 2018). Optimized strategies consist of switching to another agent, combination with different ADs, or augmentation with a different class of agents such as atypical psychotics and lithium (Chen, 2019). Practicing proper sequential treatment including initial pharmacotherapy and subsequent optimized treatment represents one of the most important and challenging issues to improve patients’ outcomes (Kim et al., 2020).

In this context, multiple international guidelines for MDD have been developed and updated continuously. Treatment strategies and alternative choices were recommended according to the evidence synthesis to achieve adequate response. Besides, appropriate trials of ADs were emphasized before next-step optimization to enable necessary and timely adjustment. Despite the wide availability and significant value of guidelines, only a small proportion of patients diagnosed with MDD received guideline-concordant treatment. A significant number of patients were not treated with first-line drugs initially (Kern et al., 2020), and patients with inadequate response were always delayed in getting treatment optimization (Herzog et al., 2017). Moreover, although most guidelines offered next-step medication options, literature on the longitudinal treatment patterns of MDD remained limited. Therefore, we have insufficient knowledge about the quality of mental health care received by patients over the course of treatment. Further research is required to depict the longitudinal patterns of medication treatment in a real-world setting and identify the gaps between guidelines and clinical practice.

Switching to another AD and combination with another class of AD were identified as optimized treatments in Chinese MDD guidelines updated in 2015 (Li et al., 2015). Clinical practice guidelines (CPGs) that have been widely promoted and recognized include the National Institute of Clinical Excellence (NICE) guidelines (NICE, 2022), American Psychiatric Association (APA) guidelines (Gelenberg et al., 2010a), Canadian Network for Mood and Anxiety Treatments (CANMAT) guidelines (Kennedy et al., 2016), World Federation of Societies of Biological Psychiatry (WFSBP) guidelines (Bauer et al., 2013), and Royal Australian and New Zealand College of Psychiatrists (RANZCP) guidelines (Malhi et al., 2015). Although most of the guidelines recommended sequencing treatments after initial inadequate response, seldom proposed a stepwise treatment regimen (algorithm) with clear recommendations for lines of treatment. A lack of practical and specific guidance based on the evidence synthesis may lead to trial-and-error process. In addition, treatment duration before next-step optimization is also needed to form instructive guidance.

Canadian Network for Mood and Anxiety Treatments (CANMAT) guidelines are recognized as authoritative guidelines and are widely embraced by health care practitioners (Gabriel et al., 2020). Characterizing by the levels of evidence and “line of treatment,” it proposed a stepwise treatment regimen (algorithm) for pharmacological treatment (Kennedy et al., 2016). The CANMAT algorithm provides operational methods to monitor treatment results and optimized therapeutic strategies to manage inadequate response to antidepressants. Unlike other antidepressant algorithms such as Japanese Psychopharmacology Algorithm Project (JPAP) algorithm and Korean Medication Algorithm Project for Depressive Disorder (KMAP-DD) algorithm with limited promotion (Motohashi et al., 2008; Seo et al., 2018), CANMAT guidelines have long been presented to Chinese psychiatrists in continuing medical education and clinical practice since the publication in 2009. As there was no paradigm of optimized treatment as well as an update on Chinese guidelines for MDD since 2015 (Feng et al., 2019b), the CANMAT algorithm has now become one of the most employed tools to support clinical decision-making in China. AD algorithm is considered as a key strategy to reduce improper and unsystematic treatment, which might be the major contributor to the pseudo-resistance that leads to poor outcomes and high medical expenditures (Ricken et al., 2018; Bauer et al., 2019). However, the concordance of the CANMAT algorithm with naturalistic antidepressant treatment patterns in China has not been studied yet. Although previous studies reported the prescribing patterns of MDD in China (Zhang et al., 2016; Chen et al., 2017; Feng et al., 2017), the longitudinal characteristics from initial pharmacotherapy to subsequent optimized treatment have not been described. Understanding the longitudinal prescription patterns especially optimized treatment strategies after the failure of monotherapy represents the first step to identify the gap between the CANMAT algorithm and real-world practice.

To fill this gap, the present study aimed to: *1*) examine the concordance between longitudinal patterns of AD treatment and the CANMAT algorithm in China; *2*) describe the discrepancies in both medication regimen and treatment duration; *3*) describe the characteristics of the algorithm-concordant/-discordant subgroups. As China has no treatment algorithm for MDD so far, we chose the CANMAT algorithm as the reference due to its synthesis of the latest evidence and wide adoption in China. Identifying inadequacies in algorithm adherence will help to promote evidence-based practice and improve the management of depression.

## Materials and methods

### Data source

This study utilized standardized electronic medical records (EMRs) database from Shanghai Mental Health Center (SMHC), which is one of the largest mental health institutions in China and provides medical services for patients with mental illness across the country. The EMRs offer a comprehensive set of patient and clinical information on demographics (gender and age), ICD-10 diagnostic codes, drug prescriptions, procedures and exact date of medical service events (diagnosis, prescription, hospital admission and discharge, *etc*.). Each individual has a unique identifier. A medical record with demographic information, psychiatric diagnosis, medical notes and prescribed agents was required to be created by the authorized psychiatrist during the hospital visit. All EMRs data were de-identified and extracted from the hospital information system (HIS) into a validated and standardized EMR database by the hospital information technology department. This retrospective database study was approved by the Institutional Review Board (IRB) of Shanghai Mental Health Center and written informed consent from patients was waived given the anonymous database.

### Study design and study population

**FIGURE 1:**
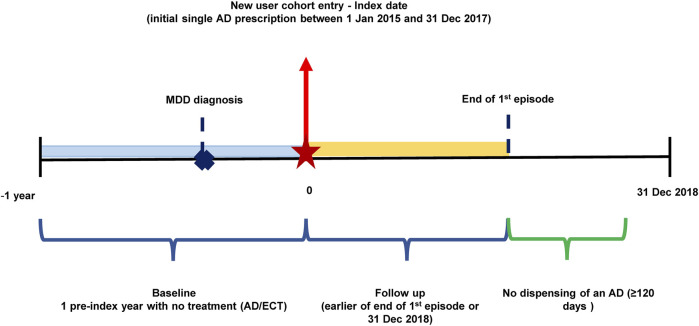
Illustration of the study design.

This is a retrospective cohort study among Chinese MDD patients who were assessed and treated at SMHC during the year 2015–2018. Patients entered the study cohort when they initiated pharmacotherapy with a single AD between 1 January 2015 and 31 December 2017 and the date of entry (date of the first AD prescription) was defined as the index date. One year free of ADs prescription record prior to the index date was required to avoid the influences of historical treatment. The baseline period was defined as 1 year prior to the index date and patients were followed up to the end of the first episode or the end of 2018, whichever came first. An episode was defined as a period when a patient exposed to at least one dispensing of AD medications. An episode ends when the patient had 120 days with no dispensing of an AD medication (Figure 1). (Fife et al., 2017; Shin et al., 2020) Patients were included in the study cohort if they met the following criteria: *1*) aged 18–64 years on the index date, *2*) diagnosed with Major Depressive Disorder (ICD-10 code: F32.x, F33.x) during the baseline period, *3*) prescribed with at least one AD during follow-up. To ensure the naturalistic treatment patterns of MDD, patients were excluded if they: *1*) received a diagnosis of bipolar (ICD-10 code: F30.x, F31.x) or schizophrenia (ICD-10 code: F20.x) during baseline or follow-up, *2*) had electroconvulsive therapy (ECT) before the index date, *3*) diagnosed with central nervous system disease(s) (ICD-10 code: F00.x- F09.x) during baseline or follow-up, *4*) patients with multiple ADs (two or more) on the index date. Initial ADs included selective serotonin reuptake inhibitors (SSRIs), serotonin and norepinephrine reuptake inhibitors (SNRIs), tricyclic ADs (TCAs), noradrenergic and specific serotonergic ADs (NaSSAs), and other ADs (e.g., agomelatine, trazodone, bupropion, reboxetine, maprotiline, mianserin). Supplementary Table S1 listed the drugs included in this study, consisting of all ADs approved in the Chinese market and available in SMHC, and ADs recommended in the CANMAT algorithm.

### Longitudinal treatment patterns, treatment level and treatment duration

**FIGURE 2:**
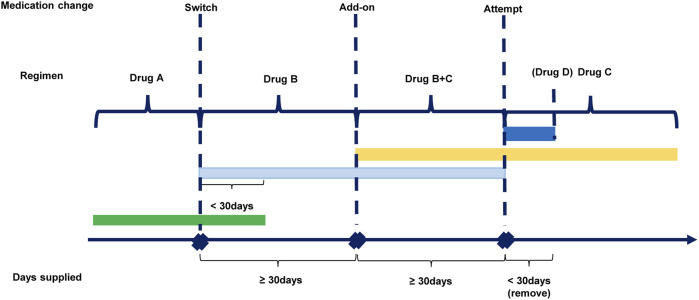
Defining different patterns of medication change.

The longitudinal pattern of medication of each patient was estimated through follow-up and patterns of medication change were defined based on clinical perspective and referred from a published database study (Kern et al., 2020). Prescription of two drugs with at least 30 days of overlap was defined as an “add-on.” Prescription of a different drug (with at least 30 days supplied) following the previous drug or with fewer than 30 days overlap was considered as a “switch” and was considered an “attempt” if the days of supply were less than 30 days. The “attempt” was observed but the drug used was not calculated since its duration is too short to be considered as a normal course of medication (Figure 2). A grace period of 14 days for the same drug was applied. The terms “treatment level” and “regimen” were used to describe the sequential medication: the index drug in the initial prescription was defined as a Level 1 regimen. If a patient’s medication change was considered as a switch or add-on compared to Level 1 (Level n) according to the above definition, then he/she entered Level 2 (Level n+1). If the change was an attempt, the patient stayed in the current level (Level n) and the drug was ineligible to be considered as a next-level (Level n+1) regimen.

Treatment duration for each treatment level refers to the length of AD drugs treatment at that level. For example, the treatment duration of level 1 was defined as the period from the first day entering the cohort to the day before entering level 2. For those patients who didn’t enter the next level, the end of treatment duration was defined as the end of follow-up.

### Definition of concordance with CANMAT algorithm

CANMAT algorithm was identified to examine the consistency of initial AD and sequencing treatment with guideline recommendations due to its explicit algorithm-based guidance and wide embracement in China (Recommendations for optimized treatment after inadequate response to initial antidepressant from different guidelines were summarized in Supplementary Table S2). Longitudinal patterns of medication treatment were considered consistent with the CANMAT algorithm if they followed the algorithm recommended by CANMAT 2016 Clinical Guidelines for the Management of Adults with Major Depressive Disorder: Section 3. Pharmacological Treatments (Kennedy et al., 2016). CANMAT revised its 2009 version to reflect the new evidence in the field in September 2016. Recommendations for the first-line antidepressants and subsequent optimized strategies in CANMAT 2009 guidelines were retained in the 2016 update, except buspirone was removed from the recommended add-on agents. The recommended regimen by treatment level is as follow (detail drugs for each level as recommended could be found in Supplementary Tables S3, S4),

Level 1: initiate with a first-line AD

Level 2/Level 3: *1*) Switch to an AD with evidence for superior efficacy or a second-line or third-line AD, or *2*) add an adjunctive medication in the recommendation list.

Concordance with the CANMAT algorithm was defined as agreement at all three levels. Adherence to the algorithm at level 1, but not level 2 or 3 would not be considered as concordant practice. This study only focused the early treatment strategy on the first three levels as it determines the early improvement which is the strong predictor of treatment outcome (Tunvirachaisakul et al., 2018).

### Statistical analysis

The demographic and clinical characteristics of eligible patients on the index date and during follow-up were described. Summary measures for categorical variables (percentages) and continuous variables (mean, standard deviation (SD); median, first and third quartile (Q1 and Q3)) were tabulated and presented. The number and proportion of algorithm-concordant treatment were calculated among total patients or sub-sample with optimized treatment (switch/add-on) after recommended first-line treatment, overall and by index year. The Chi-square test for trend (Cochran-Armitage test for trend) was used to compare the proportions between years. Drug utilization of the first three levels, including treatment regimens and treatment duration was analyzed. Demographics and health care utilization of patients who received CANMAT-concordant/-discordant treatment were described separately. Statistical analysis was conducted with SAS, version 9.4 (SAS Institute, Cary, NC, United States).

## Results

### Baseline demographic and clinical characteristics

Among the 40,856 MDD patients (ICD code: F32.X, F33.X) initiated AD prescription during 2015–2017, 38 were excluded for previous electroconvulsive therapy, 1476 were excluded for concurrent diagnosis of bipolar disorders, schizophrenia, and organ disease of the central nervous system, 6888 were excluded for not meeting age criteria and 12,697 were excluded for multi-drugs at initial prescription. Finally, a total of 19,955 eligible MDD patients who initiated pharmacotherapy with a single AD were included in the study cohort. Supplementary Figure S1 presents a flowchart describing the inclusion process. Baseline demographic and clinical characteristics are presented in Table 1. The mean age of the study cohort was 36.78 (SD = 13.01), and the largest age group was adults aged 18 to 30 (*n* = 7960; 39.89%). A large proportion of the patients were female (*n* = 13,221; 66.25%), and most (*n* = 19,862; 99.53%) were attending outpatient clinics on the index date. The majority of the patients had only one level of treatment (*n* = 17,003, 85.21%). About 10% of patients had two or three levels (two levels: 1430, 7.17%; three levels: 799, 4.00%), and only 3.62% (*n* = 723) had four levels or more. The median follow-up period of total patients and those who only had one level was 39.0 days (Q1-Q3: 20–142) and 28 days (Q1-Q3: 15–75) respectively.

**TABLE 1:** Demographic and clinical characteristics of the study population.

Characteristics	Study population (*N* = 19,955)
Age (years), mean (SD)	36.78 (13.01)
Age group, *n* (%)	
18–30	7960 (39.89)
31–40	5227 (26.19)
41–50	2861 (14.34)
51–65	3907 (19.58)
Gender, *n* (%)	
Male	6734 (33.75)
Female	13,221 (66.25)
Place of service on the index date, *n* (%)	
Outpatient	19,862 (99.53)
Inpatient	93 (0.47)
Follow-up period (days), median (Q1-Q3)	39 (20–142)
Number of prescriptions, median (Q1-Q3)	1 (1–4)
Number of visits/person-year	12.39
Number of hospitalizations/person-year	3.06

Data are presented as the percentage or the median (interquartile range) as appropriate. SD, standard deviation; Q1, first quartile; Q3, third quartile.

### Concordance with CANMAT treatment algorithm

Among 19,955 eligible patients identified, the treatment for 17,074 patients (85.56%) followed the CANMAT algorithm during follow-up, including 16,263 patients who had only one level of treatment. Of the 17 initial ADs identified in level 1, 12 were first-line drugs in the CANMAT algorithm. The concordance proportion on level 1 was 96.42% (*n* = 19,240). The concordance proportion decreased to 27.24% (*n* = 811) among those patients who moved beyond level 1. The concordance proportions by index year were tabulated in Table 2. Overall concordance proportion significantly increased from 84.45% to 86.03% during the year of 2015–2017 (Z = −2.372; *p* = 0.018), while the proportions for optimized treatment remained 26.29%, 27.14%, and 27.93 respectively (Z = −0.808; *p* = 0.419).

**TABLE 2:** Trend comparison of concordance proportions between years.

Guideline-concordance	Index year	*n*	Proportion, %	Z	*p Value*
Overall	2015	4225	84.45	−2.372	**0.018**
	2016	5847	85.82		
	2017	7002	86.03		
First-line treatment	2015	4797	95.88	−2.553	**0.011**
	2016	6569	96.42		
	2017	7874	96.74		
Optimized treatment^a^	2015	204	26.29	−0.808	0.419
	2016	269	27.14		
	2017	338	27.93		

Bold values indicate *p* < 0.1.

a Percentages were computed among the number of patients who had medication changes after recommended first-line treatment.

### Longitudinal characteristics of treatment duration

With the increase in treatment level, an extension of treatment duration was observed, and the median duration were 28, 64.5, and 70.5 days for level 1–3, respectively. For patients stayed in level 1 during the study, 24.97% stopped treatment in 2 weeks. The median treatment duration of level 2 and level 3 were more than 4 weeks, and 54.14% and 61.17% reached 8 weeks respectively. Before the first change in medication (entered level 2), 27.08% of patients had more than 8 weeks of level 1 treatment (index AD), whereas 32.10% had less than 2 weeks of treatment. Of those who entered level 2, all had more than 4 weeks of treatment and 54.14% had more than 8 weeks of treatment before entering level 3 (Table 3).

**TABLE 3:** Duration of treatment levels of study patients.

Duration of treatment level	Level 1 (*N* = 19,955)			Level 2 (*N* = 2952)			Level 3 (*N* = 1522)
	*Only index drug (n = 16,849)*	*With Medication change (n = 3106)*	*Total (n = 19,955)*	*Stopped at level 2 (n = 1430)*	*With Medication change (n = 1522)*	*Total (n = 2952)*	
Mean (SD)	87.25 (151.40)	63.87 (107.75)	83.61 (145.71)	150.20 (178.54)	102.49 (101.22)	125.6 (145.89)	141.05 (159.25)
Median (Q1-Q3)	28 (15–75)	28 (14–63)	28 (14–72)	74 (41–178)	61 (40–124)	64.5 (41–146)	70.5 (43–171)
<2 weeks, %	4208 (24.97)	997 (32.10)	5205 (26.08)	0 (0)	0 (0)	0 (0)	0 (0)
2–4 weeks, %	4728 (28.06)	736 (23.70)	5464 (27.38)	0 (0)	0 (0)	0 (0)	0 (0)
4–6 weeks, %	1945 (11.54)	323 (10.40)	2268 (11.37)	377 (26.36)	468 (30.75)	845 (28.62)	378 (24.84)
6–8 weeks, %	939 (5.57)	209 (6.73)	1148 (5.75)	199 (13.92)	230 (15.11)	429 (14.53)	213 (13.99)
>8 weeks, %	5029 (29.85)	841 (27.08)	5870 (29.42)	854 (59.72)	824 (54.14)	1678 (56.84)	931 (61.17)

SD, standard deviation; Q1, first quartile; Q3, third quartile.

### Longitudinal characteristics of medication regimen

In the study cohort (*N* = 19,955), 17 different ADs were observed as initial drugs, and the top five ADs were Escitalopram (*n* = 5861, 29.37%), Sertraline (*n* = 3573, 17.91%), Mirtazapine (*n* = 2047, 10.26%), Venlafaxine (*n* = 2018, 10.11%) and Paroxetine (*n* = 1762, 8.83%). The medication changes from level 1 to level 3 were shown in Table 4. In the study cohort, 3106 (15.57%) had at least one change in treatment regimen during follow-up and the number of patients having a switch, add-on from level 1 to level 2 was 1601 (8.02%), 1351 (6.77%) respectively. 154 (0.77%) patients attempted to change the treatment regimen stayed at level 1. Among patients who had a switch from level 1 to level 2 (*N* = 1601), the number of patients further had a switch or add-on from level 2 to level 3 was 427 (26.67%) and 220 (13.74%) respectively, and the rest 954 (59.59%) remained unchanged. The top three drugs used under the switch pattern from level 1 to level 2 were escitalopram (*N* = 179; 11.18%), venlafaxine (*N* = 135; 8.43%), and mirtazapine (*N* = 110; 6.87%). Among patients who had an add-on from level 1 to level 2 (*N* = 1351), 534 (39.53%) did not undergo further medication changes, and the number of patients who had a switch or add-on from level 2 to level 3 was 813 (60.18%) and 4 (0.30%) respectively. The top three drugs used under the add-on pattern from level 1 to level 2 were mirtazapine (*N* = 306; 22.65%), quetiapine (*N* = 212; 15.69%), and trazodone (*N* = 189; 13.99%).

**TABLE 4:** Medication changes on the first three levels of patients initiated single AD.

	*N* (%)
Patients initiated with a single AD	19,955 (100)
Total number of patients with medication change during follow-up	3106 (15.57)
Switch (Level 1 → Level 2) ^a^	1601 (8.02)
Top three drugs ^b^	
Escitalopram	179 (11.18)
Venlafaxine	135 (8.43)
Mirtazapine	110 (6.87)
Subsequent medication change ^b^	
Pattern 1a: Switch → Unchanged	954 (59.59)
Pattern 1b: Switch → Switch	427 (26.67)
Pattern 1c: Switch → Add-on	220 (13.74)
Add-on (Level 1 → Level 2) ^a^	1351 (6.77)
Top three drugs ^c^	
Mirtazapine	306 (22.65)
Quetiapine	212 (15.69)
Trazodone	189 (13.99)
Subsequent medication change ^c^	
Pattern 2a: Add-on → Unchanged	476 (39.53)
Pattern 2b: Add-on → Switch	871 (60.18)
Pattern 2c: Add-on → Add-on	4 (0.30)
Attempt (Level 1) ^a^	154 (0.77)

a Percentages were computed among the number of patients initiated with single ADs (*N* = 19,955).

b Percentages were computed among the number of patients who had a switch from level 1 to level 2 (*N* = 1601).

c Percentages were computed among the number of patients who had an add-on from level 1 to level 2 (*N* = 1351). AD, antidepressants.

### Algorithm-discordant practice from level 1 to level 2 of patients who initiated with recommended first-line AD

**FIGURE 3:**
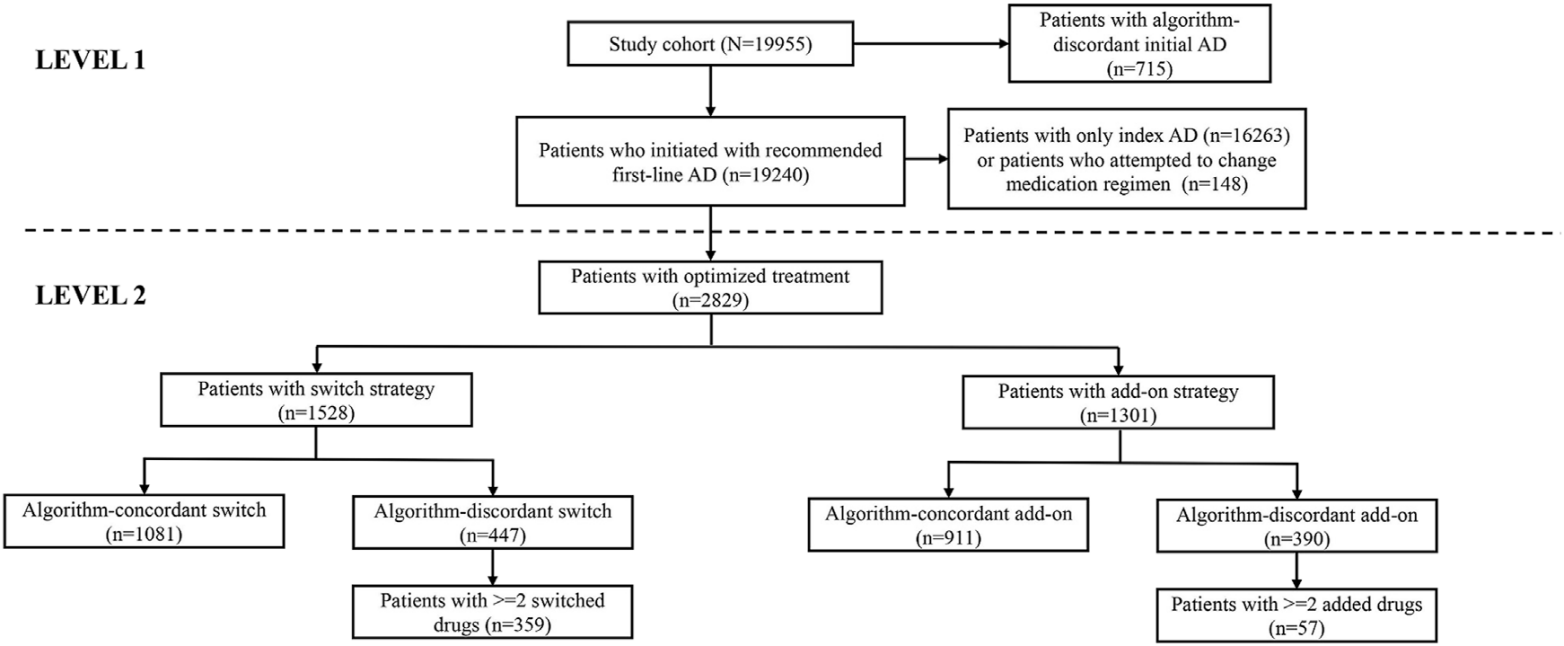
Flowchart of the algorithm-discordant practice from level 1 to level 2.

 Among 19,240 patients who initiated recommended first-line drugs, 2829 (14.70%) received subsequent optimized treatment (moved to level 2 with switch or add-on strategy). Among the 1528 patients with switch strategy, 447 (29.25%) received an algorithm-discordant switch pattern, including 359 (23.49%) with ≥ 2 switching drugs at a time. Among the 1301 patients with add-on strategy, 390 (29.98%) received an algorithm-discordant add-on pattern, including 57 (4.38%) with ≥ 2 adding drugs at a time. Figure 3 presents the flowchart of the algorithm-discordant practice from level 1 to level 2.

#### Characteristics of patients who received CANMAT algorithm-concordant/-discordant treatment after algorithm-concordant initial therapy

Among 2977 patients who initiated recommended first-line drugs and received optimized treatment, 811 patients followed CANMAT algorithm during their subsequent treatment. Demographics and healthcare utilization of algorithm-concordant/-discordant subgroup were presented in Table 5. Overall, the median follow-up duration of algorithm-concordant subgroup and algorithm-discordant subgroup was 153 (Q1-Q3 = 79–128) and 368 days (Q1-Q3 = 181–577) respectively. The average number of clinical visits per person-year was 13.07 and 13.08 among patients who received algorithm-concordant and discordant treatment respectively. The median time length between hospital visits of patients with and without algorithm-concordant treatment was 25 (Q1-Q3 = 16.17–37.88) and 29.17 (Q1-Q3 = 21.33–38.79) respectively.

**TABLE 5:** Demographics and healthcare utilization of patients who received CANMAT algorithm-concordant/-discordant treatment.

	Patients who received CANMAT algorithm-concordant treatment (*N* = 811)	Patients who received CANMAT algorithm-discordant treatment (*N* = 2166)
Age (years), mean (SD)	38.87 (13.89)	37.94 (13.92)
Gender, %		
Male	280 (34.53)	762 (35.18)
Female	531 (65.47)	1404 (64.82)
Follow-up duration (days), median (Q1-Q3)	153 (79–328)	368 (181–577)
Number of clinical visits/person-year	13.07	13.08
Intervals of hospital visits (days), median (Q1-Q3)	25.00 (16.17–37.88)	29.17 (21.33–38.79)
Duration of hospital stay (days), median (Q1-Q3)	38 (23–51)	37.5 (25.5–52.5)

SD, standard deviation; Q1, first quartile; Q3, third quartile.

## Discussion

Our study described the longitudinal characteristics of treatment patterns for MDD in China. To our knowledge, this is the first study to examine the concordance of the current practice of AD treatment with the CANMAT algorithm in a real-world setting. The overall proportion of algorithm-concordant treatment with the CANMAT algorithm was more than 80%, but only about 27% of patients received algorithm-concordant optimized treatment (switch/add-on). The gaps between algorithm recommendations and clinical practice existed in both optimized strategies and treatment duration.

The real-world practice by Chinese clinicians in the overall prescriptions was quite concordant with the CANMAT algorithm, as the overall concordance proportion reached 85.56% in this study. In our previous study on guideline concordance in the treatment of acute bipolar depression, 49.8% of patients were treated in adherence to CANMAT guidelines (Wang et al., 2014). Our results suggested that patients with unipolar depression were more likely to receive guideline-concordant treatment compared to those with bipolar depression. In addition, this disparity might be resulted from the efforts in the dissemination of evidence-based medicine and CANMAT guidelines in China. However, consistency in the present study is mainly attributed to the consensus on the first choice of ADs. When it comes to the subsequent optimized treatments, the concordance proportion was much lower and turned out to be 27.24%. Although there is an increasing yearly trend in the concordance proportion, the unsatisfied situation has not been improved for the optimized treatment. Algorithm-guided treatment is recognized as a stepwise treatment regimen for timely and efficient optimization to manage treatment-resistant depression (Bauer et al., 2019). Although adherence to algorithms may not benefit every patient, it offers general principles and systematic instructions for clinicians based on the evidence synthesis. Inconsistencies with algorithms could lead to more trial-and-error processes and reduce the remission rate of depression (Kim et al., 2020). Implementation of standardized guidelines was considered as the priority to improve the quality of medical care, especially in underdeveloped countries and regions (Lee et al., 2020). Our results suggested the urgent need for guideline-concordant practice in optimizing strategies for depression following unsatisfied outcomes of initial prescriptions. In the present study, 15.74% of patients initiated with first-line AD were identified to enter level 2. This is in contrast with the previous studies that more than 50% of patients required optimized treatment following monotherapy (Rush et al., 2006), which may be explained by the loss of follow-up and thus subsequent prescriptions were unavailable to obtain. Another explanation might be the improved treatment efficacy caused by supplementary treatment such as psychotherapy and repetitive transcranial magnetic stimulation (rTMS), which was commonly used in the hospital based on the medication treatment.

It has long been argued that what should be the ideal gap between the onset of initial ADs and optimized treatment. In the present study, treatment duration was not considered when defining the algorithm-concordant treatment. As the appropriate length of treatment is emphasized in every guideline and has a great influence on the clinical outcome, we measured the treatment duration of different levels to provide additional evidence on the algorithm discordance. Chinese guidelines recommended clinicians wait for 4–6 weeks for ADs to work before considering treatment adjustment, however, substantial evidence suggested that shortening time might result in better clinical outcomes (Kudlow et al., 2014; Habert et al., 2016). CANMAT algorithm proposed to consider optimized treatment at 2–4 weeks after initial pharmacotherapy. Evidence showed that improvement in the first 2 weeks was a significant predictor for later remission (Wagner et al., 2017; Hicks et al., 2019). However, our results showed that over 30% of patients underwent medication changes in the first 2 weeks after initial monotherapy. This premature move may be inappropriate as low-quality evidence supported the optimization within 2 weeks after initial pharmacotherapy unless followed by tolerability problems (Olgiati et al., 2018). Notably, none of the level 2 treatment regimens was optimized within 4 weeks, indicating that the psychiatrist preferred to maintain the treatment regimen for a longer time if it has already been optimized once. We found that 27.8% and 54.14% of patients maintained the level 1 and level 2 treatment regimen for more than 8 weeks respectively before entering the next level. One possible reason is the adherence to other clinical guidelines such as American Psychiatric Association (APA) guideline (Gelenberg et al.). APA guideline recommends an adequate trial up to 8 weeks, which seems outdated given the recent evidence. It has been suggested that delaying effective treatment could lead to poor outcomes and MDD patients should be treated with optimized treatment urgently (Romera et al., 2012; Habert et al., 2016; Kraus et al., 2019). Moreover, for patients with prior medication, early optimized treatment at 2 weeks showed a superior treatment effect (Dreimüller et al., 2019). Our results suggested that early optimized treatment especially for inadequate response to level 2 treatment regimen should be emphasized to improve treatment outcomes.

Both Chinese and CANMAT guidelines for MDD included first-line recommendations for physicians to select and initiate the ADs. In the present study, SSRIs, SNRIs and NaSSAs–the new generation of ADs as the first recommendations in both Chinese and CANMAT guidelines (Kennedy et al., 2016; Feng et al., 2019a), were the most prescribed initiated ADs. This is consistent with the previous studies conducted in China and many other regions (Dold et al., 2016; Zhang et al., 2016; Gauthier et al., 2017), suggesting that the initiators recommended by guidelines were widely adopted by clinicians. As for subsequent optimizing pharmacotherapy, we found escitalopram, venlafaxine and mirtazapine were the top three choices for switching strategies after initial treatment. This is in accordance with the first-line recommendations in the CANMAT algorithm. For add-on strategies, although mirtazapine was recommended as the second-line adjunctive medication, approximately 23% of patients used mirtazapine in combination with the initial AD under add-on patterns. The combination treatment of mirtazapine and venlafaxine is referred to as “California rocket fuel” due to its advantages in efficacy and fast-acting. This treatment regimen was proposed in 2009, when a study found that the Remeron-Effexor had the greatest overall benefit for MDD patients among the combinations of ADs with Remeron (Blier et al., 2010). However, the evidence for “California rocket fuel” turned out to be flimsy, as subsequent studies failed to reproduce this result (Henssler et al., 2016; Kessler et al., 2018). In the latest Chinese treatment guidelines for MDD published in 2015, mirtazapine and trazodone were the first recommendations for combination as an alternative when switching strategy has failed (Li et al., 2015). Our result is consistent with a recent UK study that found mirtazapine was the most commonly prescribed AD and mostly used in a combination with venlafaxine (Paton et al., 2020), suggesting that this combination is still a preferred choice despite the contradictory evidence. Our results showed that trazodone was the third most frequently used drug in add-on strategies, although it was recommended as the third-line adjunctive medication in CANMAT guidelines given its association with the poor response rate and side effects burden (Andrade, 2018). In addition to the impact of published Chinese guidelines, another possible reason is the sedative effects of trazodone which has been widely recognized and applied in the treatment of depressed patients with sleep disturbances (Treviño et al., 2017; Settimo and Taylor, 2018). Although the combination of mirtazapine with initial AD was still the mainstream regimen, our study showed a trend in prescribing adjunctive antipsychotics, especially quetiapine, indicating that Chinese psychiatrists were adapting their clinical decisions to the up-to-date evidence and guidelines when considering adjunctive agents for poor response.

For the optimized treatment regimen after initial AD, our results showed that one-third of patients had the algorithm-discordant switch strategy and add-on strategy respectively. For algorithm-discordant switch, most (80.3%) substitute the initial medication with two new drugs at a time. It suggested that the discordant practice in switch strategy was mainly due to the aggressive adjustment of the regimen. Clinicians prefered to go a step further than algorithm recommendations, prescribing combination of other drugs rather than just substituting the initial AD. Alternatively, 14.62% of algorithm-discordant add-on strategy consists of two new adding drugs, especially combination of ADs, which were not included in the add-on recommendation list, were the most used drugs in the algorithm-discordant add-on strategy. This is in accordance with the previous studies that combination of two antidepressants was still widely recognized and used among clinicians despite its relatively slight effect and potential side effects compared to antipsychotic augmentation (Gobbi et al., 2018; Gronemann et al., 2021). Clinicians should remain aware of and alert to the drug interactions of antidepressants.

Our results showed a shorter median length of follow-up among the patients who received guideline-concordant treatment compared with the discordant subgroup. This is reasonable as the guideline concordance was mainly attributed to the consensus of the initiated ADs. The guideline-concordant subgroup was mostly composed of patients who only stayed in level 1 and had a shorter follow-up period compared to those who entered level 2. In addition, algorithm-discordant treatment could lead to improper clinical practice that could cause resistance or recurrent course of depression (Wang et al., 2019). Patients might switch to local or primary medical facilities such as community hospitals after significant improvement or remission. Further research with rigorous experimental methods is required to validate the results and examine the concordance-related factors.

There are several limitations of this study. Firstly, we only included patients with a previous 1 year of no AD prescription, aiming to include patients who initiated pharmacotherapy for comparison with the algorithm from the first-line treatment. However, patients may not be completely “new user” of AD treatment as their medical history outside the study hospital was not captured. Further, only patients initiated with ADs were included. The extent and profile of overall medications like hypnotics, anticonvulsants, and stimulants were not described thus some inappropriate or irrational prescriptions could not be evaluated. Secondly, this study was likely to overestimate the concordance proportion as it focused on the medication regimens on the first three levels and dose-escalation was not considered, which need to be further examined. In this study, we described the duration of treatment levels to obtain a general understanding of its consistency with the recommendations in CANMAT guidelines. Also, some psychotic depression patients prescribed AD could not be well distinguished, however, based on baseline diagnosis, the proportion of such patients was quite low and had limit impact on the main results of this study. Thirdly, this was a descriptive study mainly focusing on the discrepancies between AD algorithm and clinical practice. Although the characteristics of the algorithm-concordant/-discordant subgroup were described, no statistical test was conducted to detect significant differences. Future research and group comparison methods are required to explore the factors associated with the algorithm-adherence practice. Finally, our data were derived from a single tertiary mental health institution. Although medical referral was not compulsory, the study population consisted of a high proportion of patients with recurrent depression, treatment resistance, and complex conditions referred from the second or primary hospitals. Cautions should be taken when extrapolating the results to other institutions as specialists might turn their backs on the general recommendations to achieve individual treatment. However, the patients who visited our institute came from different provinces and regions, which enhanced the demographic generalizability of the results. There might be measurement bias as some patients visited other hospitals and received medications during their follow-up period, which was not reflected in our EMR database. A considerable proportion of patients who stayed at the first level were lost to follow-up, thus their subsequent medication was unavailable for concordance assessment. Loss to follow-up in the database may be due to improvement in disease, treatment failure, moving to other hospitals or cities, and many other reasons. Future research is required to describe the overall extent of naturalistic medication based on the integrated database. Meanwhile, this study aimed to identify the gap between physicians’ practice and guideline recommendations to promote guideline practice on the doctors’ level. Factors related to patients’ adherence to the prescriptions should be included to examine the algorithm-concordant practice in the future.

To summarize, although the overall concordance proportion seemed relatively satisfactory, this concordance was mostly attributed to the initial pharmacotherapy and decreased dramatically during the subsequent optimized treatment. Gaps existed between clinical practice and algorithm, mostly and notably in improper adjunctive strategies and inadequate or extended duration of treatment before optimization. These gaps need to be bridged to improve the quality of medical care and promote evidence-based medicine. Our results indicated that there is still room to improve algorithm-based clinical practice in China, especially for patients who needed treatment optimizations.

## Data Availability

The original contributions presented in the study are included in the article/Supplementary Material, further inquiries can be directed to the corresponding authors.

## References

[B1] Andrade C. (2018). How to read a research paper: An exercise in critical thinking in the context of an epidemiologic study on tamsulosin and the risk of dementia. J. Clin. Psychiatry 79 (2), 18f12660. 10.4088/JCP.18f12660 30549500

[B2] Bauer M. Pfennig A. Severus E. Whybrow P. C. Angst J. Moeller H.-J. (2013). World federation of Societies of biological Psychiatry (WFSBP) guidelines for biological treatment of unipolar depressive disorders, part 1: Update 2013 on the acute and continuation treatment of unipolar depressive disorders. World J. Biol. Psychiatry 14 (5), 334–385. 10.3109/15622975.2013.804195 23879318

[B3] Bauer M. Rush A. J. Ricken R. Pilhatsch M. Adli M. (2019). Algorithms for treatment of major depressive disorder: Efficacy and cost-effectiveness. Pharmacopsychiatry 52 (3), 117–125. 10.1055/a-0643-4830 29986372

[B4] Bayes A. J. Parker G. B. (2018). Comparison of guidelines for the treatment of unipolar depression: A focus on pharmacotherapy and neurostimulation. Acta Psychiatr. Scand. 137 (6), 459–471. 10.1111/acps.12878 29577229

[B5] Bergfeld I. O. Mantione M. Figee M. Schuurman P. R. Lok A. Denys D. (2018). Treatment-resistant depression and suicidality. J. Affect. Disord. 235, 362–367. 10.1016/j.jad.2018.04.016 29665520

[B6] Blier P. Ward H. E. Tremblay P. Laberge L. Hébert C. Bergeron R. (2010). Combination of antidepressant medications from treatment initiation for major depressive disorder: A double-blind randomized study. Am. J. Psychiatry 167 (3), 281–288. 10.1176/appi.ajp.2009.09020186 20008946

[B7] Chen P. (2019). Optimized treatment strategy for depressive disorder. Adv. Exp. Med. Biol. 1180, 201–217. 10.1007/978-981-32-9271-0_11 31784965

[B8] Chen Y. Bennett D. Clarke R. Guo Y. Yu C. Bian Z. (2017). Patterns and correlates of major depression in Chinese adults: A cross-sectional study of 0.5 million men and women. Psychol. Med. 47 (5), 958–970. 10.1017/s0033291716002889 27919307 PMC5341494

[B9] Dold M. Kautzky A. Bartova L. Rabl U. Souery D. Mendlewicz J. (2016). Pharmacological treatment strategies in unipolar depression in European tertiary psychiatric treatment centers - a pharmacoepidemiological cross-sectional multicenter study. Eur. Neuropsychopharmacol. 26 (12), 1960–1971. 10.1016/j.euroneuro.2016.10.005 27816317

[B10] Dreimüller N. Wagner S. Engel A. Braus D. F. Roll S. C. Elsner S. (2019). Predictors of the effectiveness of an early medication change strategy in patients with major depressive disorder. BMC Psychiatry 19 (1), 24. 10.1186/s12888-019-2014-x 30642308 PMC6332626

[B11] Emslie G. J. Ventura D. Korotzer A. Tourkodimitris S. (2009). Escitalopram in the treatment of adolescent depression: A randomized placebo-controlled multisite trial. J. Am. Acad. Child. Adolesc. Psychiatry 48 (7), 721–729. 10.1097/CHI.0b013e3181a2b304 19465881

[B12] Feng Y. Sha S. Hu C. Wang G. Ungvari G. S. Chiu H. F. (2017). Prescribing patterns of psychotropic medications and clinical features in patients with major depressive disorder with and without comorbid dysthymia in China. Asia-Pacific Psychiatry 9 (1), e12261. 10.1111/appy.12261 27759189

[B13] Feng Y. Xiao L. Wang W.-W. Ungvari G. S. Ng C. H. Wang G. (2019a). Guidelines for the diagnosis and treatment of depressive disorders in China: The second edition. J. Affect. Disord. 253, 352–356. 10.1016/j.jad.2019.04.104 31078835

[B14] Feng Y. Xiao L. Wang W. W. Ungvari G. S. Ng C. H. Wang G. (2019b). Guidelines for the diagnosis and treatment of depressive disorders in China: The second edition. J. Affect. Disord. 253, 352–356. 10.1016/j.jad.2019.04.104 31078835

[B15] Fife D. Feng Y. Wang M. Y. Chang C. J. Liu C. Y. Juang H. T. (2017). Epidemiology of pharmaceutically treated depression and treatment resistant depression in Taiwan. Psychiatry Res. 252, 277–283. 10.1016/j.psychres.2017.03.006 28288438

[B16] Gabriel F. C. de Melo D. O. Fráguas R. Leite-Santos N. C. Mantovani da Silva R. A. Ribeiro E. (2020). Pharmacological treatment of depression: A systematic review comparing clinical practice guideline recommendations. PLoS One 15 (4), e0231700. 10.1371/journal.pone.0231700 32315333 PMC7173786

[B17] Gauthier G. Guérin A. Zhdanava M. Jacobson W. Nomikos G. Merikle E. (2017). Treatment patterns, healthcare resource utilization, and costs following first-line antidepressant treatment in major depressive disorder: A retrospective US claims database analysis. BMC Psychiatry 17 (1), 222. 10.1186/s12888-017-1385-0 28629442 PMC5477263

[B18] Gelenberg A. J. Freeman M. P. Markowitz J. C. Rosenbaum J. F. Thase M. E. Trivedi M. H. (2010b). Practice guideline for the treatment of patients with major depressive disorder. Virginia, US: American Psychiatric Association.

[B19] Gelenberg A. J. Freeman M. Markowitz J. Rosenbaum J. Thase M. Trivedi M. (2010a). American Psychiatric Association practice guidelines for the treatment of patients with major depressive disorder. Am. Psychiatric Assoc. 167 (10), 9–118.

[B20] Gobbi G. Ghabrash M. F. Nuñez N. Tabaka J. Di Sante J. Saint-Laurent M. (2018). Antidepressant combination versus antidepressants plus second-generation antipsychotic augmentation in treatment-resistant unipolar depression. Int. Clin. Psychopharmacol. 33 (1), 34–43. 10.1097/YIC.0000000000000196 28906325

[B21] Gronemann F. H. Petersen J. Alulis S. Jensen K. J. Riise J. Ankarfeldt M. Z. (2021). Treatment patterns in patients with treatment-resistant depression in Danish patients with major depressive disorder. J. Affect. Disord. 287, 204–213. 10.1016/j.jad.2021.03.029 33799039

[B22] Habert J. Katzman M. A. Oluboka O. J. McIntyre R. S. McIntosh D. MacQueen G. M. (2016). Functional recovery in major depressive disorder: Focus on early optimized treatment. Prim. Care Companion CNS Disord. 18 (5). 10.4088/PCC.15r01926 27835721

[B23] Henssler J. Bschor T. Baethge C. (2016). Combining antidepressants in acute treatment of depression: A meta-analysis of 38 studies including 4511 patients. Can. J. Psychiatry. 61 (1), 29–43. 10.1177/0706743715620411 27582451 PMC4756602

[B24] Herzog D. P. Wagner S. Ruckes C. Tadic A. Roll S. C. Härter M. (2017). Guideline adherence of antidepressant treatment in outpatients with major depressive disorder: A naturalistic study. Eur. Arch. Psychiatry Clin. Neurosci. 267 (8), 711–721. 10.1007/s00406-017-0798-6 28421334

[B25] Hicks P. B. Sevilimedu V. Johnson G. R. Tal I. Chen P. Davis L. L. (2019). Predictability of nonremitting depression after first 2Weeks of antidepressant treatment: A VAST‐D trial report. Psychiatr. Res. Clin. Pract. 1 (2), 58–67. 10.1176/appi.prcp.20190003 PMC9176018 36101874

[B26] Kennedy S. H. Lam R. W. McIntyre R. S. Tourjman S. V. Bhat V. Blier P. (2016). Canadian Network for mood and anxiety treatments (CANMAT) 2016 clinical guidelines for the management of adults with major depressive disorder: Section 3. Pharmacological treatments. Can. J. Psychiatry. 61 (9), 540–560. 10.1177/0706743716659417 27486148 PMC4994790

[B27] Kern D. M. Cepeda M. S. Defalco F. Etropolski M. (2020). Treatment patterns and sequences of pharmacotherapy for patients diagnosed with depression in the United States: 2014 through 2019. BMC Psychiatry 20 (1), 4. 10.1186/s12888-019-2418-7 31900133 PMC6942399

[B28] Kessler D. S. MacNeill S. J. Tallon D. Lewis G. Peters T. J. Hollingworth W. (2018). Mirtazapine added to SSRIs or SNRIs for treatment resistant depression in primary care: Phase III randomised placebo controlled trial (MIR). Bmj 363, k4218. 10.1136/bmj.k4218 30381374 PMC6207929

[B29] Kim J. M. Stewart R. Kang H. J. Kim J. W. Lee H. J. Jhon M. (2020). Short and long-term treatment outcomes of stepwise psychopharmacotherapy based on early clinical decision in patients with depressive disorders. J. Affect. Disord. 274, 315–325. 10.1016/j.jad.2020.05.002 32469822

[B30] Kraus C. Kadriu B. Lanzenberger R. Zarate C. A. Jr. Kasper S. (2019). Prognosis and improved outcomes in major depression: A review. Transl. Psychiatry 9 (1), 127. 10.1038/s41398-019-0460-3 30944309 PMC6447556

[B31] Kudlow P. A. McIntyre R. S. Lam R. W. (2014). Early switching strategies in antidepressant non-responders: Current evidence and future research directions. CNS Drugs 28 (7), 601–609. 10.1007/s40263-014-0171-5 24831418

[B32] Lee Y. Brietzke E. Cao B. Chen Y. Linnaranta O. Mansur R. B. (2020). Development and implementation of guidelines for the management of depression: A systematic review. Bull. World Health Organ. 98 (10), 683–697h. 10.2471/blt.20.251405 33177758 PMC7652558

[B33] Li L. J. Ma X. Fang Y. R. Si T. M. Wang G. Xu X. F. (2015). Practice guideline for prevention and treatment of depression disorder (in Chinese). Beijing.

[B34] Malhi G. S. Bassett D. Boyce P. Bryant R. Fitzgerald P. B. Fritz K. (2015). Royal Australian and New Zealand College of Psychiatrists clinical practice guidelines for mood disorders. Aust. N. Z. J. Psychiatry 49 (12), 1087–1206. 10.1177/0004867415617657 26643054

[B35] Motohashi N. Shioe K. Nakamura J. Ohshima A. Yamada K. Ozawa H. (2008). Revised psychopharmacological algorithms for the treatment of mood disorders in Japan. Int. J. Psychiatry Clin. Pract. 12 (1), 11–18. 10.1080/13651500701330791 24916491

[B36] NICE (2022). Depression in adults: Treatment and management NICE guideline [NG222]. London: National Institute for Health and Care Excellence. 35977056

[B37] Olgiati P. Serretti A. Souery D. Dold M. Kasper S. Montgomery S. (2018). Early improvement and response to antidepressant medications in adults with major depressive disorder. Meta-analysis and study of a sample with treatment-resistant depression. J. Affect. Disord. 227, 777–786. 10.1016/j.jad.2017.11.004 29254066

[B38] Paton C. Anderson I. M. Cowen P. J. Delgado O. Barnes T. R. E. (2020). Prescribing for moderate or severe unipolar depression in patients under the long-term care of UK adult mental health services. Ther. Adv. Psychopharmacol. 10, 2045125320930492. 10.1177/2045125320930492 32595931 PMC7297128

[B39] Ricken R. Wiethoff K. Reinhold T. Stamm T. J. Baghai T. C. Fisher R. (2018). A standardized stepwise drug treatment algorithm for depression reduces direct treatment costs in depressed inpatients - results from the German Algorithm Project (GAP3). J. Affect. Disord. 228, 173–177. 10.1016/j.jad.2017.11.051 29253683

[B40] Romera I. Pérez V. Menchón J. M. Schacht A. Papen R. Neuhauser D. (2012). Early vs. conventional switching of antidepressants in patients with MDD and moderate to severe pain: A double-blind randomized study. J. Affect. Disord. 143 (1-3), 47–55. 10.1016/j.jad.2012.05.024 22858211

[B41] Rossom R. C. Solberg L. I. Vazquez-Benitez G. Whitebird R. R. Crain A. L. Beck A. (2016). Predictors of poor response to depression treatment in primary care. Psychiatr. Serv. 67 (12), 1362–1367. 10.1176/appi.ps.201400285 27417890 PMC5133141

[B42] Rush A. J. Trivedi M. H. Wisniewski S. R. Nierenberg A. A. Stewart J. W. Warden D. (2006). Acute and longer-term outcomes in depressed outpatients requiring one or several treatment steps: A STAR*D report. Am. J. Psychiatry 163 (11), 1905–1917. 10.1176/ajp.2006.163.11.1905 17074942

[B43] Seo J. S. Bahk W. M. Wang H. R. Woo Y. S. Park Y. M. Jeong J. H. (2018). Korean medication algorithm for depressive disorders 2017: Third revision. Clin. Psychopharmacol. Neurosci. 16 (1), 67–87. 10.9758/cpn.2018.16.1.67 29397669 PMC5810446

[B44] Settimo L. Taylor D. (2018). Evaluating the dose-dependent mechanism of action of trazodone by estimation of occupancies for different brain neurotransmitter targets. J. Psychopharmacol. 32 (1), 96–104. 10.1177/0269881117742101 29332554

[B45] Shin D. Kim N. W. Kim M. J. Rhee S. J. Park C. H. K. Kim H. (2020). Cost analysis of depression using the national insurance system in South Korea: A comparison of depression and treatment-resistant depression. BMC Health Serv. Res. 20 (1), 286. 10.1186/s12913-020-05153-1 32252762 PMC7137426

[B46] Treviño L. A. Ruble M. W. Treviño K. Weinstein L. M. Gresky D. P. (2017). Antidepressant medication prescribing practices for treatment of major depressive disorder. Psychiatr. Serv. 68 (2), 199–202. 10.1176/appi.ps.201600087 27691378

[B47] Trivedi M. H. Rush A. J. Wisniewski S. R. Nierenberg A. A. Warden D. Ritz L. (2006). Evaluation of outcomes with citalopram for depression using measurement-based care in STAR*D: Implications for clinical practice. Am. J. Psychiatry 163 (1), 28–40. 10.1176/appi.ajp.163.1.28 16390886

[B48] Tundo A. de Filippis R. Proietti L. (2015). Pharmacologic approaches to treatment resistant depression: Evidences and personal experience. World J. Psychiatry 5 (3), 330–341. 10.5498/wjp.v5.i3.330 26425446 PMC4582308

[B49] Tunvirachaisakul C. Gould R. L. Coulson M. C. Ward E. V. Reynolds G. Gathercole R. L. (2018). Predictors of treatment outcome in depression in later life: A systematic review and meta-analysis. J. Affect. Disord. 227, 164–182. 10.1016/j.jad.2017.10.008 29100149

[B50] Wagner S. Engel A. Engelmann J. Herzog D. Dreimüller N. Müller M. B. (2017). Early improvement as a resilience signal predicting later remission to antidepressant treatment in patients with Major Depressive Disorder: Systematic review and meta-analysis. J. Psychiatr. Res. 94, 96–106. 10.1016/j.jpsychires.2017.07.003 28697423

[B51] Wang Z. Gao K. Hong W. Xing M. Wu Z. Chen J. (2014). Guidelines disconcordance in acute bipolar depression: Data from the national bipolar mania pathway survey (BIPAS) in mainland China. PLoS One 9 (4), e96096. 10.1371/journal.pone.0096096 24763748 PMC3999095

[B52] Wang Z. Ma X. Xiao C. (2019). “Standardized treatment strategy for depressive disorder,” in Depressive disorders: Mechanisms, measurement and management. Editor Fang Y. (Singapore: Springer Singapore), 193–199.

[B53] World Health Organization (2020). Depression. Geneva, Switzerland: World Health Organization.

[B54] Zhang L. Chen Y. Yue L. Liu Q. Montgomery W. Zhi L. (2016). Medication use patterns, health care resource utilization, and economic burden for patients with major depressive disorder in Beijing, People's Republic of China. Neuropsychiatr. Dis. Treat. 12, 941–949. 10.2147/ndt.s97407 27143895 PMC4844449

