# Supplementary figures and images for: Concordance of the treatment patterns for major depressive disorders between the Canadian Network for Mood and Anxiety Treatments (CANMAT) algorithm and real-world practice in China
Source: Front Pharmacol. 2022 Aug 31;13:954973. doi: 10.3389/fphar.2022.954973 (PMC9471191; doi:10.3389/fphar.2022.954973)

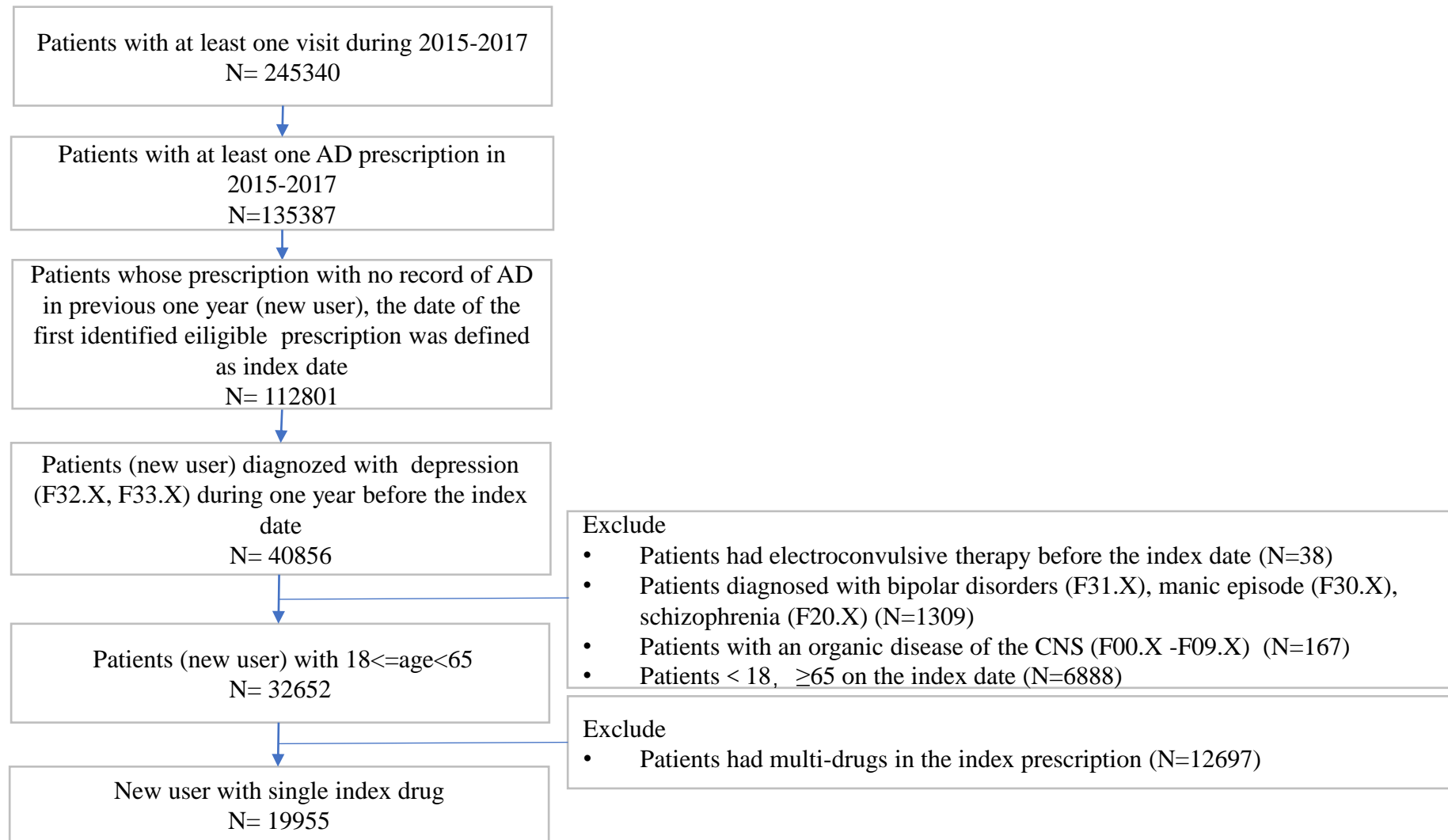

**Figure S1. Flowchart of patient selection**

Supplement: Supplementary file 1 [file DataSheet2.pdf]
