# Supplementary material for: Concordance of the treatment patterns for major depressive disorders between the Canadian Network for Mood and Anxiety Treatments (CANMAT) algorithm and real-world practice in China
Source: Front Pharmacol. 2022 Aug 31;13:954973. doi: 10.3389/fphar.2022.954973 (PMC9471191; doi:10.3389/fphar.2022.954973)
Supplement: Supplementary file 2 [file DataSheet1.docx]

**Supplementary materials**

**Table S1.** Drug list of the drugs included in the study

| **Drug class** | **Antidepressants** |
| --- | --- |
| Selective serotonin reuptake inhibitors (SSRIs) | Escitalopram, Citalopram, Fluoxetine, Fluvoxamine, Paroxetine, Sertraline |
| Serotonin and noradrenalin reuptake inhibitors (SNRIs) | Duloxetine, Milnacipran, Venlafaxine |
| Noradrenergic and specific serotonergic antidepressants (NaSSA) | Mirtazapine |
| Tricyclic antidepressants (TCAs) | Amitriptyline, Clomipramine, Doxepin, Imipramine, Tianeptine |
| Other antidepressants (other ADs) | Bupropion, Reboxetine, Trazodone, Maprotiline, Mianserin，Agomelatine |
| Antipsychotics and Lithium (others) | Aripiprazole, Quetiapine, Risperidone, Lithium, Olanzapine, Methylphenidate, Ziprasidone |

**Table S2.** Recommendations for optimized treatment after inadequate response to initial antidepressant from different guidelines

| Guidelines | Treatment duration of initial AD | pharmacotherapeutic strategies |
| --- | --- | --- |
| CANMAT 2016 guidelines (Kennedy et al., 2016) | No early improvement (> 20%-30% reduction in scores on a depression rating scale) within 2-4 weeks | Details can be seen in Table S3 |
| CANMAT 2009 guidelines (Lam et al., 2009) | Little improvement (< 20% improvement in scores on a depression rating scale) within 2 weeks.  Wait for another 2–4 weeks before considering additional strategies for those who have more than minimal improvement (≥ 20% improvement in scores on a depression rating scale) within 4-6 weeks | 1. Dose increase 2. Switch and add-on strategies with recommendations for lines of treatment.   *Lines of treatment and level of evidence were updated in 2016.  *Buspirone was removed from the add-on recommendations in 2016 update. |
| Chinese 2015 guidelines (Li et al., 2015) | No response within 4-6 weeks | 1. switching to another antidepressant in the same or different class 2. Combining with another class of antidepressants if the switch strategy has failed |
| NICE guidelines (NICE, 2009)  NICE CG90 | 1. Absent or minimal response within 3-4 weeks of treatment with therapeutic dose of an antidepressant 2. Inadequate response within another 2-4 weeks after 4-week treatment with some improvement | 1. Dose increase 2. Switching to another antidepressant 3. Combining with another antidepressant (such as mirtazapine or mianserin) 4. Augmenting with lithium or antipsychotic 5. (NICE CG90) |
| NICE guidelines (NICE, 2022)  NICE NG222 | No response within 4-6 weeks at a recognized therapeutic dose | 1. Further-line treatment strategies include dose escalation, switching to drugs in the same or different class, combination with a different class of antidepressant or a second-generation antipsychotic, and augmenting antidepressants with lamotrigine or triiodothyronine |
| APA guidelines (Gelenberg et al., 2010) | No improvement or minimal improvement in symptoms within 4-8 weeks | 1. Dose increase 2. Augmenting the antidepressant with other agents 3. Changing to another non-MAOI antidepressant |
| WFSBP 2013 guidelines (Bauer et al., 2013) | Insufficient response within 2 weeks | 1. Dose increase 2. Switching to another antidepressant in the same or different class 3. Combining two antidepressants from different classes 4. Augmenting the antidepressant with other agents (e.g., lithium, thyroid hormone, or atypical antipsychotics)   *augmentation with lithium, quetiapine, and aripiprazole were the most recommended. |
| RANZCP 2015 guidelines (Malhi et al., 2015) | No improvement within the first three weeks of adequate treatment | 1. Dose increase 2. Combining antidepressants 3. Augmenting with lithium and/or antipsychotic medication 4. Switch strategy (only be considered under an adequate trial at an adequate dose) |
| MOH 2012 guidelines (Lim and Ling, 2012) | All antidepressants should be continued for 4-6 weeks at least | 1. Dose increase 2. Switching within the class or to a different class of antidepressant 3. Lithium augmentation and thyroid hormone augmentation |

*Abbreviations*: AD, antidepressant. CANMAT, Canadian Network for Mood and Anxiety Treatments. NICE, National Institute for Health and Clinical Excellence. APA, American Psychiatric Association. WFSBP, World Federation of Societies of Biological Psychiatry. RANZCP, Royal Australian and New Zealand College of Psychiatrists. MOH, The Ministry of Health.

**Brief introduction of Japan and Korean algorithm**

***Japanese Psychopharmacology Algorithm Project (JPAP) algorithm***

Algorithm for the treatment of mild or moderate major depressive disorder from Japan recommended SSRIs (fluvoxamine and paroxetine) and an SNRI (milnacipran) as first-line treatment. If treatment is not effective (considering dosage and duration), a switch to another antidepressant or augmentation strategies should be considered, and Lithium is recommended as the most effective drug for augmentation. The whole algorithm figure below is referred from published paper (Motohashi et al., 2008).


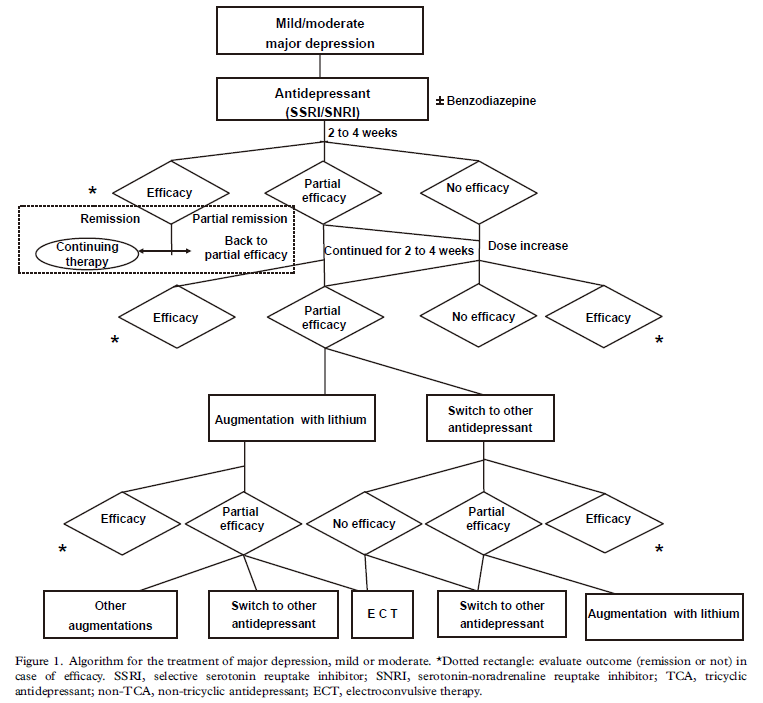


***Korean Medication Algorithm Project for Depressive Disorder (KMAP-DD) algorithm***

The Korean Medication Algorithm for Major Depressive Disorder was developed and issued by the KMAP-DD in 2002 and has been updated constantly, with first revision conducted in 2006, second revision in 2012 and third revision in 2017. The algorithm in 2017 was similar to that of 2012 version, but the preference of atypical antipsychotics (AAPs) was more increased. For mild-to-moderate depressive episodes, AD monotherapy was recommended as the first-line strategy. The augmentation of AD＋AAP was also the preferred (first-line) strategy, which indicated the increased preference for AAP. Based on the patients response to initial strategies, switching and adding AD or AAP were recommended for the 2nd step. The whole algorithm is shown in the figure below, referred from published paper (Seo et al., 2018).


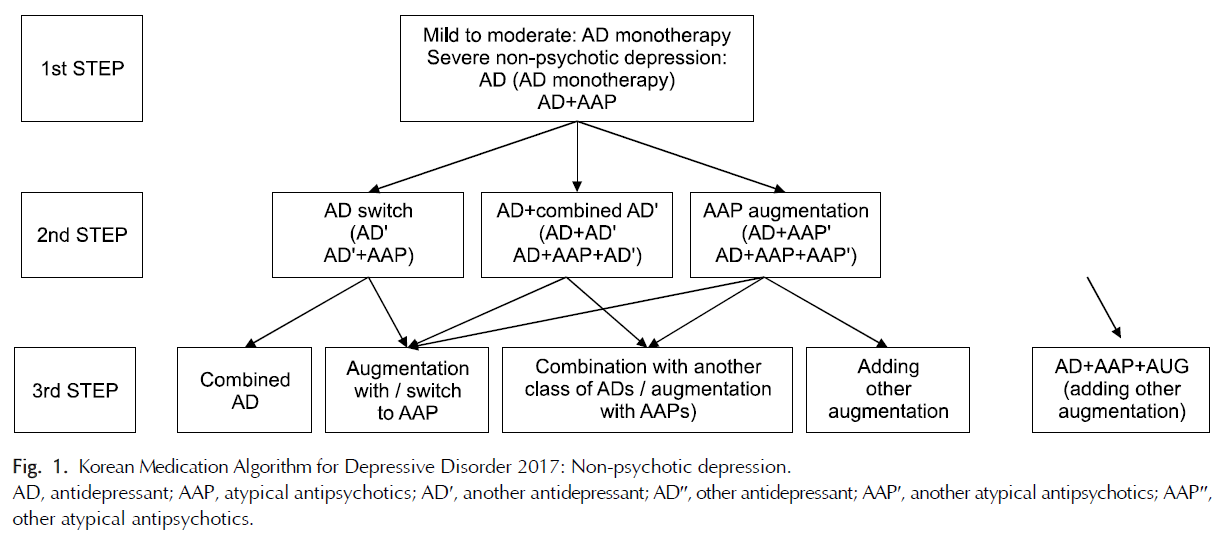


***Detail drugs in definition of concordance with CANMAT algorithm***

As recommended in CANMAT algorithm, the recommended treatment level can be briefly summarized as below, with the drugs recommended listed (some drugs recommended in guideline but not available in SMHC are not listed).


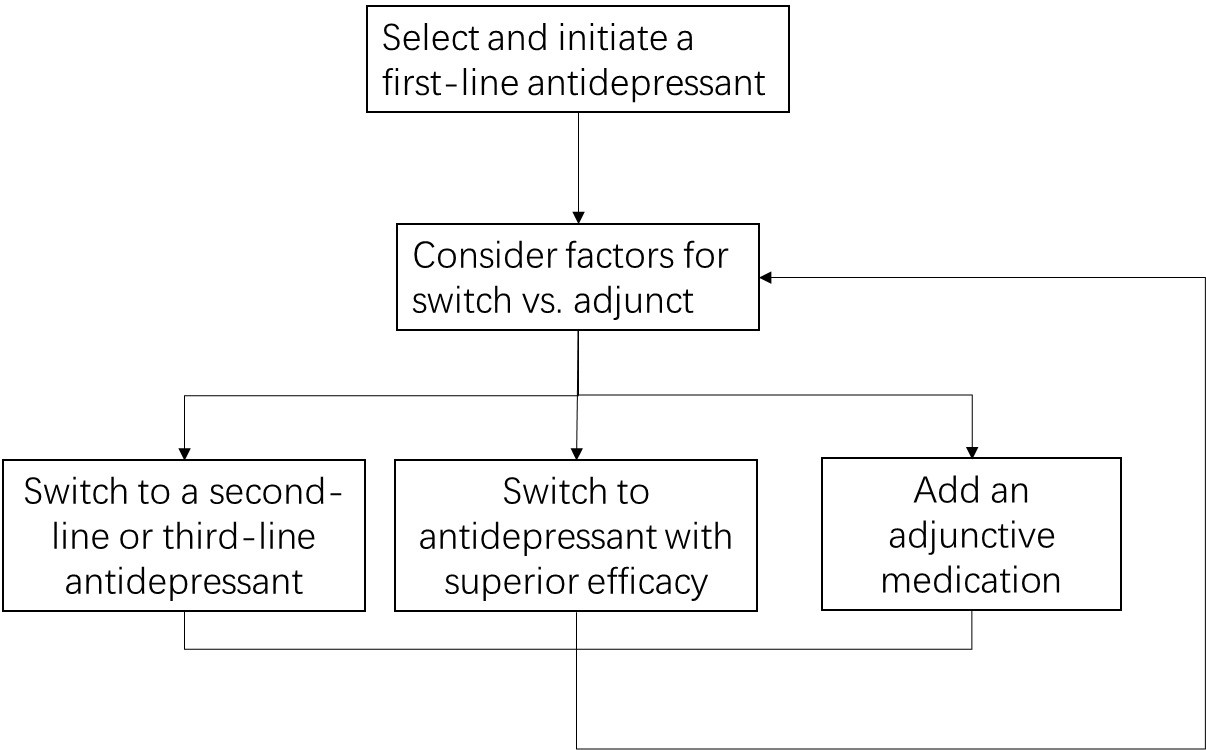


**Table S3.** Brief introduction of CANMAT algorithm and drugs recommended

| CANMAT algorithm recommendation |
| --- |
| Level 1: initiate with a first-line antidepressant  Level2/level3:(1) switch to a second-line or third-line antidepressant  (2) switch to antidepressant with superior efficacy  (3) add an adjunctive medication |
| First-line antidepressant: |
| Escitalopram, Citalopram, Duloxetine, Mirtazapine, Paroxetine, Sertraline, , Venlafaxine, Agomelatine, Bupropion, Fluoxetine, Fluvoxamine, Mianserin |
| Second-line and third-line antidepressants: |
| TCA (Amitriptyline, Clomipramine, Doxepin, Imipramine, Tianeptine), Quetiapine, Trazodone, Reboxetine |
| Adjunctive medication: |
| Aripiprazole, Quetiapine, Risperidone, Bupropion, Mirtazapine, Mianserin, Ziprasidone, Lithium, Olanzapine, Methylphenidate |
| Superior efficacy drugs: |
| Escitalopram vs. Citalopram, duloxetine, fluoxetine, fluvoxamine, paroxetine  Mirtazapine vs. Duloxetine, fluoxetine, fluvoxamine, paroxetine, sertraline, venlafaxine  Sertraline vs. Duloxetine, fluoxetine, fluvoxamine, paroxetine  Venlafaxine vs. Duloxetine, fluoxetine, fluvoxamine, paroxetine  Agomelatine vs. Fluoxetine, sertraline  Citalopram vs. Paroxetine |

Thus, based on the algorithm recommendation, the drugs in below scenario were considered concordant with the algorithm.

**Table S4.** Detail drugs of levels considered concordant with CANMAT algorithm

| Level 1 | Level 2/level3 | | |
| --- | --- | --- | --- |
|  | Switch | | Add-on |
| First-line drugs | Second/third line drugs | Superior efficacy drugs | Adjunctive medication |
| Escitalopram | Amitriptyline  Clomipramine  Doxepin  Imipramine  Tianeptine  Quetiapine  Trazodone  Reboxetine | / | Aripiprazole  Quetiapine Risperidone  Bupropion  Mirtazapine  Mianserin  Ziprasidone  Lithium  Olanzapine  Methylphenidate |
| Citalopram |  | Escitalopram |  |
| Duloxetine |  | Mirtazapine  Sertraline  Venlafaxine |  |
| Mirtazapine |  | / |  |
| Paroxetine |  | Escitalopram  Mirtazapine  Sertraline  Venlafaxine  Citalopram |  |
| Sertraline |  | Mirtazapine  Agomelatine |  |
| Trazodone |  | / |  |
| Venlafaxine |  | Mirtazapine |  |
| Agomelatine |  | / |  |
| Bupropion |  | / |  |
| Fluoxetine |  | Escitalopram  Mirtazapine  Sertraline  Venlafaxine  Agomelatine |  |
| Fluvoxamine |  | Escitalopram  Mirtazapine  Sertraline  Venlafaxine |  |
| Mianserin |  | / |  |

**References**

Bauer, M., Pfennig, A., Severus, E., Whybrow, P.C., Angst, J., Moeller, H.-J., et al. (2013). World Federation of Societies of Biological Psychiatry (WFSBP) guidelines for biological treatment of unipolar depressive disorders, part 1: update 2013 on the acute and continuation treatment of unipolar depressive disorders. 14(5)**,** 334-385.

Gelenberg, A.J., Freeman, M., Markowitz, J., Rosenbaum, J., Thase, M., Trivedi, M., et al. (2010). American Psychiatric Association practice guidelines for the treatment of patients with major depressive disorder. 167(Suppl 10)**,** 9-118.

Kennedy, S.H., Lam, R.W., McIntyre, R.S., Tourjman, S.V., Bhat, V., Blier, P., et al. (2016). Canadian Network for Mood and Anxiety Treatments (CANMAT) 2016 Clinical Guidelines for the Management of Adults with Major Depressive Disorder: Section 3. Pharmacological Treatments. *Can J Psychiatry* 61(9)**,** 540-560. doi: 10.1177/0706743716659417.

Lam, R.W., Kennedy, S.H., Grigoriadis, S., McIntyre, R.S., Milev, R., Ramasubbu, R., et al. (2009). Canadian Network for Mood and Anxiety Treatments (CANMAT) Clinical guidelines for the management of major depressive disorder in adults.: III. Pharmacotherapy. 117**,** S26-S43.

Li, L.J., Ma, X., Fang, Y.R., Si, T.M., Wang, G., Xu, X.F., et al. (2015). *Practice Guideline For Prevention and Treatment of Depression Disorder (in Chinese).* Beijing.

Lim, E., and Ling, J.J.S.M.J. (2012). Ministry of Health clinical practice guidelines: Depression. 53(2)**,** 137-144.

Malhi, G.S., Bassett, D., Boyce, P., Bryant, R., Fitzgerald, P.B., Fritz, K., et al. (2015). Royal Australian and New Zealand College of Psychiatrists clinical practice guidelines for mood disorders. 49(12)**,** 1087-1206.

Motohashi, N., Shioe, K., Nakamura, J., Ohshima, A., Yamada, K., Ozawa, H., et al. (2008). Revised psychopharmacological algorithms for the treatment of mood disorders in Japan. *Int J Psychiatry Clin Pract* 12(1)**,** 11-18. doi: 10.1080/13651500701330791.

NICE (2009). Depression in adults: recognition and management. NICE guideline (CG 90).

NICE (2022). *Depression in adults: treatment and management NICE guideline [NG222]* [Online]. London: National Institute for Health and Care Excellence. Available: <https://www.nice.org.uk/guidance/ng222> [Accessed 12 July 2022].

Seo, J.S., Bahk, W.M., Wang, H.R., Woo, Y.S., Park, Y.M., Jeong, J.H., et al. (2018). Korean Medication Algorithm for Depressive Disorders 2017: Third Revision. *Clin Psychopharmacol Neurosci* 16(1)**,** 67-87. doi: 10.9758/cpn.2018.16.1.67.
